# Calcium phosphate particles stimulate interleukin-1β release from human vascular smooth muscle cells: A role for spleen tyrosine kinase and exosome release

**DOI:** 10.1016/j.yjmcc.2017.12.007

**Published:** 2018-02

**Authors:** Yana Dautova, Alexander N. Kapustin, Kevin Pappert, Matthias Epple, Hanneke Okkenhaug, Simon J. Cook, Catherine M. Shanahan, Martin D. Bootman, Diane Proudfoot

**Affiliations:** a Signalling Programme, Babraham Institute, Babraham, Cambridge CB22 3AT, UK; b Cardiovascular Division, James Black Centre, King's College London,125 Coldharbour Lane, London SE5 9NU, UK; c Inorganic Chemistry and Center for Nanointegration Duisburg-Essen (CeNIDE), University of Essen-Duisburg, Essen 45117, Germany; d School of Life, Health and Chemical Sciences, The Open University, Milton Keynes MK7 6AA, UK

**Keywords:** Vascular smooth muscle, Calcium phosphate particles, Cytokines, Caspase-1, SYK, Exosomes, CaP, calcium phosphate, IL-1β, interleukin-1β, VSMC, vascular smooth muscle cells, SYK, spleen tyrosine kinase, LPS, lipopolysaccharide, BM, basal culture medium, SFM, serum-free medium, MSU, monosodium urate, ATP, adenosine triphosphate, NLRP3: nucleotide-binding domain, leucine-rich repeat/pyrin domain-containing-3

## Abstract

**Aims:**

Calcium phosphate (CaP) particle deposits are found in several inflammatory diseases including atherosclerosis and osteoarthritis. CaP, and other forms of crystals and particles, can promote inflammasome formation in macrophages leading to caspase-1 activation and secretion of mature interleukin-1β (IL-1β). Given the close association of small CaP particles with vascular smooth muscle cells (VSMCs) in atherosclerotic fibrous caps, we aimed to determine if CaP particles affected pro-inflammatory signalling in human VSMCs.

**Methods and results:**

Using ELISA to measure IL-1β release from VSMCs, we demonstrated that CaP particles stimulated IL-1β release from proliferating and senescent human VSMCs, but with substantially greater IL-1β release from senescent cells; this required caspase-1 activity but not LPS-priming of cells. Potential inflammasome agonists including ATP, nigericin and monosodium urate crystals did not stimulate IL-1β release from VSMCs. Western blot analysis demonstrated that CaP particles induced rapid activation of spleen tyrosine kinase (SYK) (increased phospho-Y525/526). The SYK inhibitor R406 reduced IL-1β release and caspase-1 activation in CaP particle-treated VSMCs, indicating that SYK activation occurs upstream of and is required for caspase-1 activation. In addition, IL-1β and caspase-1 colocalised in intracellular endosome-like vesicles and we detected IL-1β in exosomes isolated from VSMC media. Furthermore, CaP particle treatment stimulated exosome secretion by VSMCs in a SYK-dependent manner, while the exosome-release inhibitor spiroepoxide reduced IL-1β release.

**Conclusions:**

CaP particles stimulate SYK and caspase-1 activation in VSMCs, leading to the release of IL-1β, at least in part via exosomes. These novel findings in human VSMCs highlight the pro-inflammatory and pro-calcific potential of microcalcification.

## Highlights

• CaP particles induce IL-1β release from human VSMCs.
• Senescent cells display higher basal and CaP-stimulated IL-1β release.
• Inflammasome agonists ATP, nigericin and MSU crystals do not induce IL-1β release from human VSMCs.
• CaP particle-induced IL-1β release is dependent on SYK, caspase-1 and exosome release.

## Graphical abstract

**Figure:**
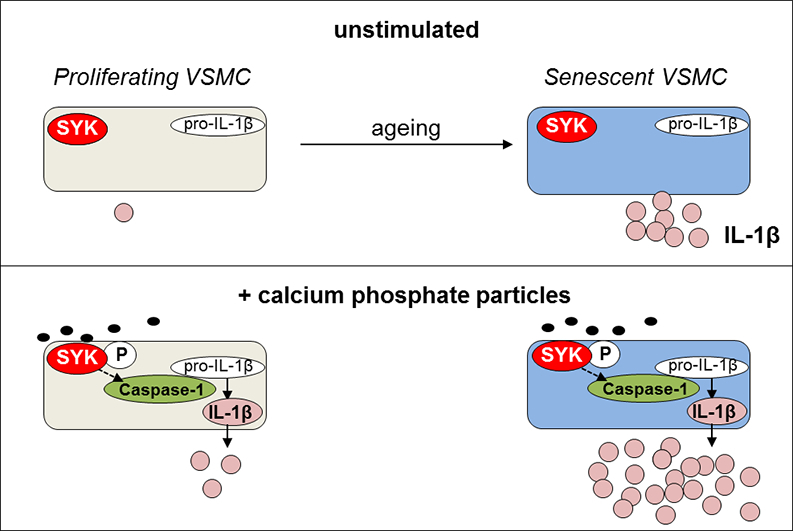

## 1 Introduction

Vascular calcification is a regulated process occurring in aging and diseased blood vessels that correlates positively with cardiovascular deaths. The calcific deposits consist of bone-like nanoparticles that often form aggregates of various sizes and degrees of crystallinity. In particular, the very small calcium phosphate (CaP) particles that appear as ‘speckled’ deposits in human atherosclerotic plaques are associated with plaque instability, increasing the likelihood of plaque rupture and subsequent thrombus formation [1], [2], [3]. Recent studies suggest that small CaP particles may be damaging due to effects on mechanical stress in the fibrous cap [4], [5], or that CaP particles can be engulfed by macrophages leading to secretion of pro-inflammatory cytokines [6]. Our own studies suggest that small CaP particles are taken up into vascular smooth muscle cells (VSMCs) via endocytosis, macropinocytosis and plasma membrane damage, causing transient intracellular Ca^2+^ rises and inducing cell death in subsets of VSMCs [7], [8]. Serum proteins, particularly fetuin-A, inhibit cell death by binding CaP particles and delaying particle uptake, membrane damage and dissolution. CaP particles are implicated in the pathogenesis of other diseases including severe calcification of skin arterioles seen in calciphylaxis, degenerative arthritis and breast cancer [9], [10], [11], [12]. The association of calcification with inflammation in vivo either in joints or in blood vessels could be explained by several in vitro studies where small particles of CaP were found to interact with neutrophils, macrophages and dendritic cells, subsequently activating various signalling mechanisms that lead to the release of pro-inflammatory cytokines [6], [13], [14], [15].

IL-1β has been shown to have a key function in the recruitment of neutrophils in vivo in response to monosodium urate (MSU) crystals [16], cholesterol crystals [17] and CaP crystals [18], [19]. IL-1β secretion generally requires a two-step process where cells first synthesise pro-IL-1β, followed by a second stimulus to cleave pro-IL-1β to its active form by inflammasome-activated caspase-1, although other proteases such as neutral elastase can also cleave pro-IL-1β [20]. The steps involved in crystal interaction with cells leading to the activation of caspase-1 are not fully understood, however, SYK activation, intracellular Ca^2+^ increases, phosphorylation of ASC (apoptosis associated speck-like protein containing a CARD) and K^+^ efflux have been reported to occur upstream of caspase-1 activation in various cell types [21], [22]. SYK is most commonly known for its role in coupling immune recognition receptors bearing cytoplasmic immunoreceptor tyrosine-based activation motifs (ITAMs) to intracellular signalling pathways but also has roles in cancer, autophagy and in pro-inflammatory responses to calcified particles in osteoarthritic joints [23], [24], [25].

VSMCs reside in the media of blood vessels and their function is to maintain vascular tone by co-ordinated contraction and dilation. VSMCs are known to display considerable phenotypic plasticity and migrate from the medial layer into the intima of diseased blood vessels where they become proliferative and secrete extracellular matrix that contributes to the strength of the fibrous cap that overlies the soft atherosclerotic plaque, protecting it from rupture. In addition to VSMCs acquiring osteoblast, chondrocyte or adipocyte-like features, VSMCs secrete several pro-inflammatory cytokines constitutively as the cells age and become senescent [26], [27], [28], [29]. Furthermore, VSMCs were recently shown to differentiate to a macrophage-like phenotype in a mouse in vivo model of atherosclerosis [30].

VSMCs are the main cell type within the fibrous cap region that overlies atherosclerotic plaques, which is thought to form as part of the healing process and to protect the underlying necrotic core from exposure to the blood vessel lumen. Small particles of CaP at this location are associated with plaque rupture in man and are thought to reflect active formation of microcalcification [3], [31], [32], [33], [34], [35], making the exploration of CaP particle effects on human VSMCs clinically relevant. With reports that human VSMCs express caspase-1 [36] and that various forms of crystals can activate caspase-1 in several cell types, we aimed to determine if CaP particles could influence human VSMC pro-inflammatory mediator production. We identified a SYK-dependent pathway in VSMCs leading to caspase-1 activation and release of IL-1β via exosomes after exposure to CaP particles. Our study highlights the pro-inflammatory potential of human VSMCs in an environment where cells are exposed to small CaP particles.

## 2 Methods

### 2.1 Cell culture

VSMCs were derived from medial layers of normal human aortae and cultured in smooth muscle basal medium (BM, Lonza) containing 5% foetal bovine serum, insulin, human fibroblast growth factor, human epidermal growth factor and gentamycin, buffered with 3.7 mg/mL NaHCO_3_ and 5% CO_2_. Some of the medial VSMCs used in this study were kindly provided by Prof. Martin Bennett (Addenbrooke's Hospital, Cambridge). Donors gave written informed consent for tissue samples to be used for research, on a standard hospital consent form. Ethical approval for use of human VSMC cultures was approved by the Cambridgeshire 1 Research Ethics Committee which conforms to the principles outlined in the Declaration of Helsinki. In some experiments, human aortic VSMCs purchased from Lonza or Promocell were used. Cells from 8 different individuals were used in this study; 4 males (aged 22, 28, 30 and 58 years old) and 4 females (aged 35, 43, 51 and 63 years old). Cells were used between passages 6 and 13 and cultured in BM, unless otherwise indicated.

### 2.2 Materials

CaP particles were prepared as described previously [8]. Briefly, aqueous solutions of calcium lactate (9.0 mM; Merck) and diammonium hydrogen phosphate (5.4 mM; Merck) were adjusted to pH 8.0 with NaOH and sterile filtered through a Filtropur S plus unit (0.2 μm). The precipitation reaction was achieved by rapidly pumping (5 mL/min) both solutions into a glass vessel under sterile conditions. The prepared suspensions were immediately centrifuged at 900 rpm for 3 min. The supernatant was removed and the particles were resuspended in 200 times less water volume than the initial nanoparticle dispersion. The concentration of Ca^2+^ in the synthesised CaP particles was quantified by atomic absorption spectroscopy (AAS; M-Serie, Thermo Electron) and particle concentrations used in this manuscript are expressed as mg/mL in terms of Ca^2+^ content, rather than weight of the nanoparticles. Under the assumption of the stoichiometry of hydroxyapatite for calcium phosphate, Ca_5_(PO_4_)_3_OH, the content of calcium phosphate can be computed as w(CaP) = w(Ca^2+^) M(Ca_5_(PO_4_)_3_OH) / 5 M(Ca) = w(Ca^2+^) 2.51. The morphology of freshly prepared nanoparticles was characterised with scanning electron microscopy (SEM; ESEM Quanta 400 FEG, gold/palladium sputtering). The particle diameter was between 30 and 60 nm. The particles had a spherical shape after the initial synthesis. Nanoparticle preparations were stored in sterile water at 4 °C and under these conditions, within 2 days the particles changed their shape (or ‘ripened’, presumably by Ostwald ripening) from a spherical to a needle-like shape. The particles were not colloidally dispersable due to the absence of a surface functionalisation (i.e. a bare, non-coated surface) and therefore agglomerated. Dynamic light scattering in pure water is impossible due to agglomeration. However, we expect a considerable enhancement of the dispersibility in cell-culture media due to the presence of proteins (formation of a stabilizing protein corona). An image displaying the needle-shaped (ripened) CaP particles interacting with human VSMCs is shown in the Supplement Fig. S1 and further examples are presented in our previous studies [8]. The zeta potential of bare calcium phosphate nanoparticles is slightly negative at neutral pH and will become more negative at increasing pH (and more positive at decreasing pH). All assays were performed with particles stored for at least 2 days. Particles were checked for sterility using an LPS/endotoxin testing kit (stocks of particles had < 0.5 EU/mL endotoxin, Pierce). Particle solutions were vortexed immediately prior to addition to cells to achieve a moderate degree of dispersion.

LPS, nigericin, ATP, and CaCl_2_ were from Sigma and Na_2_HPO_4_ was from VWR. Z-YVAD-FMK was from BioVision. MSU crystals were from Adipogen. R406 was from Selleck, and sc-202721 (spiroepoxide inhibitor) was from Insight Biotechnology. All chemicals were prepared and stored following manufacturer recommendations.

### 2.3 Propidium iodide uptake

An assay using propidium iodide (PI) as a measure of cell death was used as described previously [8].

### 2.4 ELISA

Human VSMCs in culture were exposed to various conditions in the presence or absence of CaP particles and cell supernatants were collected and stored at − 20 °C until required for analysis. Samples were defrosted, centrifuged at 13,000 rpm before measuring human IL-1β content using a bead-based Alphalisa (Perkin Elmer) or plate-based multiplex ELISA (Mesoscale discovery).

### 2.5 Preparation of cell extracts and Western blotting

Cells were lysed in ice-cold TG lysis buffer (Tris-HCl, pH 7.4, 20 mM, Triton X-100, 1% (v/v), Glycerol 10% (v/v), NaCl (137 mM), EGTA (1 mM) and MgCl_2_ (1.5 mM), NaF (0.05 M), Na_3_VO_4_ (1 mM), Aprotinin (5 μg/mL) Leupeptin (10 μg/mL) and PMSF (1 mM) and tested for protein content by BCA assay (Thermo Scientific). SDS-loading buffer was added to lysates, vortexed and heated at 95 °C for 5 min before centrifugation at 13,000 rpm and fractionation by SDS-PAGE. Cell supernatants (conditioned medium), were collected on ice into tubes containing PMSF (final concentration 1 mmol/L). SDS-loading buffer was then added and samples were incubated at 95 °C for 5 min before centrifugation at 13,000 rpm and fractionation by SDS-PAGE. Briefly, SDS-PAGE gels were transferred to Immobilon P membranes (Millipore), which were blocked in 5% (w/v) powdered milk containing 0.1% (v/v) Tween-20–TBS (tris-buffered saline) and probed with either antibodies recognising pro-IL-1β, SYK (total), phosphorylated SYK (Tyr 525/526) (all from Cell Signalling Technologies), IL-1β (R and D, AF-201), caspase-1 p20 (Adipogen), GAPDH or α-tubulin (both from ProSci). Mouse anti-human CD63 antibody was from BD Pharmingen. HRP-conjugated antibodies recognising primary antibodies were purchased from BioRad. Immune complexes were visualised using the ECL system (GE Healthcare or high sensitivity substrate, Fisher Scientific). GAPDH or α-tubulin were used as loading controls and were applied directly to re-probe original blots. In some experiments, Western blot signal intensities were quantified using ImageJ.

### 2.6 β-galactosidase staining

VSMCs were cultured in 12-well plates and assessed for β-galactosidase staining following the protocol of Chen et al [37].

### 2.7 Caspase-1 activity

Cells were treated with or without CaP particles for up to 16 h. Culture medium was then replaced with a fluorescent caspase-1 inhibitor peptide, FAM-YVAD-FLICA, for 1 h at 37 °C following the manufacturer's recommended protocol (FAM-FLICA caspase-1 assay kit, ImmunoChemistry Technologies). Cells were then washed and allowed to incubate for 1 h in basal culture medium to allow non-bound reagent to elute from cells. Cells were then stained for 5 min with Hoechst 33342 and PI (both 1 μg/mL) before placing in physiological buffer [7]. Active caspase-1 was assessed qualitatively in cells cultured in 12-well plates on a Zeiss Microbeam microscope and images were processed using ImageJ. Quantitative measurement of active caspase-1 was achieved using cells cultured in glass-bottomed black 96-well plates using an IN Cell 6000 high content screening system. Autofluorescence values for each treatment were corrected by measuring fluorescence in cells with no FAM-FLICA or no PI. Scatter plots in GraphPad were used to display levels of active caspase-1 per cell.

### 2.8 Immunofluorescence

Cells were grown on sterile glass coverslips, treated with CaP particles and exposed to FAM-YVAD-FLICA as above (section 2.7). After allowing non-bound reagent to elute for 1 h, cells were fixed in 4% paraformaldehyde, permeabilised in 0.2% Triton X/PBS, blocked for 1 h in 10% Chemibloc/0.1% Triton X/PBS, washed with 0.1% Triton X/PBS before incubating with a mouse anti-human IL-β antibody (0.25 μg/mL, R and D Systems MAB601) for 3 h. Cells were then washed in 0.1% Triton X/PBS before incubation with anti-mouse IgG NL557 conjugate (1:200 dilution, R and D Systems NL007), washed with 0.1% Triton X/PBS followed by a wash with PBS, before finally mounting in DAPI-containing hard-set mounting medium (Vectashield). Cells were imaged using a Nikon A1R confocal microscope and images were processed using ImageJ.

### 2.9 Exosome isolation and quantification

Quantification of exosomes in VSMC conditioned media was performed by using and anti-CD63-coated bead capturing assay, as described previously [38]. Briefly, anti-CD63 antibody (BD Bioscience) was immobilised on 4 μm aldehyde-sulphate latex beads (Invitrogen). VSMCs were plated on 24-well plates (10,000 cells/well) and incubated for 16 h. The cells were then washed with EBSS and incubated in M199 supplemented with 2.5% exosome-free FBS, 100 U/mL penicillin, 100 μg/mL streptomycin and 2 mM l-glutamine in the presence or absence of R406 (1 μM) for 2 h. Media were then replaced with fresh media with or without the addition of CaP particles (12.5 μg/mL) and cells were cultured for a further 16 h. Conditioned media were collected and centrifuged at 2500 x g for 5 min. The supernatants were incubated with 1 μl of anti-CD63-coated beads on a shaker overnight at 4 °C. Cells were washed twice in EBSS, trypsinised and quantified using a Nucleocounter NC-3000 (ChemoMetec A, Denmark). Anti-CD63-coated beads were washed with blocking buffer (PBS containing 2% BSA) and incubated with CD81-PE antibody (1:50 in blocking buffer) for 1 h. Next, anti-CD63-coated beads were washed with blocking buffer and analysed by flow cytometry (BD Accuri™ C6, BD Biosciences). Arbitrary units were calculated as (mean fluorescence units x percentage of positive beads) and normalised to the number of live VSMCs.

Exosomes were purified from VSMC-conditioned media by differential centrifugation as previously described [38]. In brief, VSMCs were incubated in the absence or presence of CaP particles (12.5 μg/mL) in M199 supplemented with 2.5% exosome-free FBS, 100 U/mL penicillin, 100 μg/mL streptomycin and 2 mM l-glutamine for 16 h. Next, conditioned media were collected and centrifuged for 5 min at 2500 rpm (Thermo Scientific Heraeus Multifuge 3SR + centrifuge, rotor Sorvall 75,006,441 K) and 30 min at 10,000 ×* g* (Sorvall RC6). Exosomes were pelleted from the supernatants by ultracentrifugation at 100,000 ×* g* for 40 min at 4 °C (Beckman Coulter Optima Max). Exosomes were washed with PBS, centrifuged again at 100,000 ×* g* and resuspended in PBS.

To prepare cell lysates for comparisons with exosome lysates, VSMCs were washed with PBS and lysed in 0.1 M Tris buffer (pH 8.1) supplemented with 0.15 M NaCl, 1% triton X-100 and protease inhibitor cocktail (1:100, Sigma). Cell lysates were sonicated for 5 s (Branson Sonifier 150) and centrifuged at 16,353 ×* g* for 15 min.

### 2.10 Statistics

Relevant statistical analyses were performed using GraphPad Prism and specific statistical tests are detailed in each of the Figure legends. For active caspase-1 measurements in live cells, SPSS was used to perform statistical analysis between cells from different individuals.

## 3 Results

### 3.1 CaP particles induce IL-1β release from human VSMCs

**Fig. 1:**
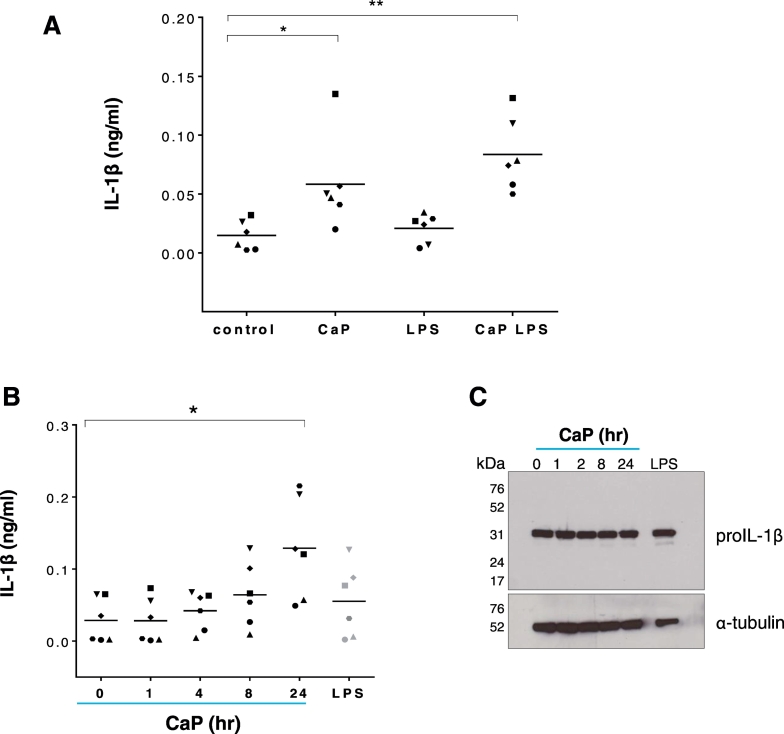
CaP particles stimulate IL-1β release. A. ELISA measurement of IL-1β release from 6 different human VSMC isolates (indicated by 6 different symbols) over 16 h with or without CaP particles (12.5 μg/mL) and with or without a 24-h pre-treatment with LPS (0.1 μg/mL). The control contained equivalent PBS. Values with means are displayed and one-way ANOVA followed by Holm-Sidak's multiple comparisons test was used to compare groups with the control. Significant differences are indicated as * (P < 0.05) or ** (P < 0.001). B. IL-1β release from 6 different human VSMC isolates exposed to CaP particles (12.5 μg/mL) at time points up to 24 h or LPS (0.1 μg/mL) for 24 h. Values with means are displayed and were analysed by one-way ANOVA followed Holm-Sidak's multiple comparisons test; significant differences from the no additions ‘0’ control are indicated as * (P < 0.01). C. Western analysis of pro-IL-1β levels in VSMC lysates treated as in B. In C, blots are representative of 4 independent experiments in cell isolates from different individuals. Results from 3 further VSMC isolates are displayed in the Supplement Fig. S2B.

To establish if CaP particles influenced IL-1β release from human VSMCs, cells were exposed to CaP particles for 16 h and IL-1β levels were measured in cell supernatants. CaP particles stimulated IL-1β release in the presence or absence of LPS, compared with controls (Fig. 1A). We confirmed that CaP particles induced release of the mature form of IL-1β (Supplement Fig. S2A). IL-1β accumulated in conditioned media after exposure to CaP particles (Fig. 1B), while cellular pro-IL-1β levels did not change after CaP particle exposure (Fig. 1C and Supplement Figs. S2B and S2C). These data suggest that pro-IL-1β is constitutively expressed in human VSMCs and that CaP particles stimulated processing and release of IL-1β, rather than stimulating de novo IL-1β synthesis. CaP particles also stimulated release of TNFα, IL-6, IL-8, DPPIV (CD26), GM-CSF and emmprin (CD147) (Supplement Fig. S3). However, we focussed the present study on the release of IL-1β from VSMCs.

**Fig. 2:**
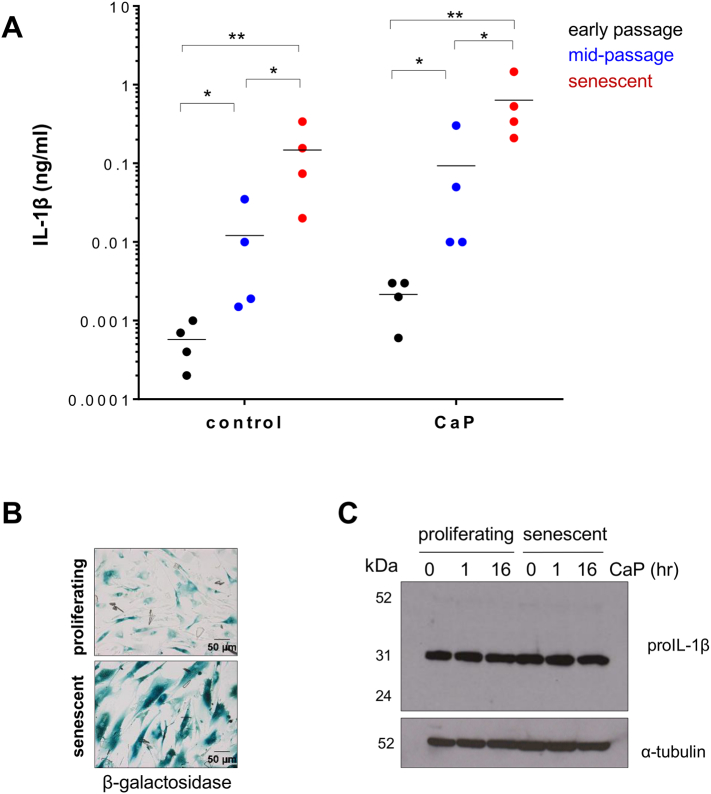
IL-1β release at different levels at different stages of VSMC culture. A. ELISA measurement of IL-1β release from early passage (3–5), mid-passage (6–13) or senescent VSMCs (14–18) with or without CaP particles (12.5 μg/mL) incubated over 16 h. Data are displayed on a log-scale and mean levels for each treatment are indicated. Data were log transformed prior to statistical analysis. A two-way ANOVA followed by a Holm-Sidak's multiple comparisons test was used to compare groups. Significant differences are indicated by * (P < 0.01) or ** (P < 0.001). 4 different VSMC isolates were used for each cell culture stage. B. β-galactosidase staining of proliferating (mid-passage) or senescent VSMCs, showing higher expression in senescent cells. C. Western analysis of pro-IL-1β levels in proliferating or senescent VSMC lysates treated with CaP particles (12.5 μg/mL) for indicated times. Blots showing results from 3 different cell isolates are shown in the Supplement Fig. S2C and S2D.

During the course of this study, it became clear that VSMCs derived from different individuals had variations in levels of IL-1β release (Fig. 1A and B). Additionally, we observed an increase in basal and CaP particle-stimulated IL-1β levels as cells matured in culture, i.e. from early passage (3–5) to mid-passage (6–13) to cells that had undergone replicative senescence (passage 14–18) (Fig. 2A); senescence was confirmed by β-galactosidase staining (Fig. 2B). No changes in cellular levels of pro-IL-1β were observed in response to CaP particles or between proliferating and senescent cells (Fig. 2C, Supplement Fig. S2C and S2D). Together, these results suggest that (i) CaP particles stimulate the processing and release of IL-1β from VSMCs and (ii) senescent VSMCs constitutively release IL-1β and exhibit a greater release of IL-1β than proliferating VSMCs in response to CaP particles. Thus, variations observed in IL-1β release from VSMCs were not only donor-dependent but also due to replicative aging in culture. Nevertheless, addition of CaP particles increased IL-1β release.

**Fig. 3:**
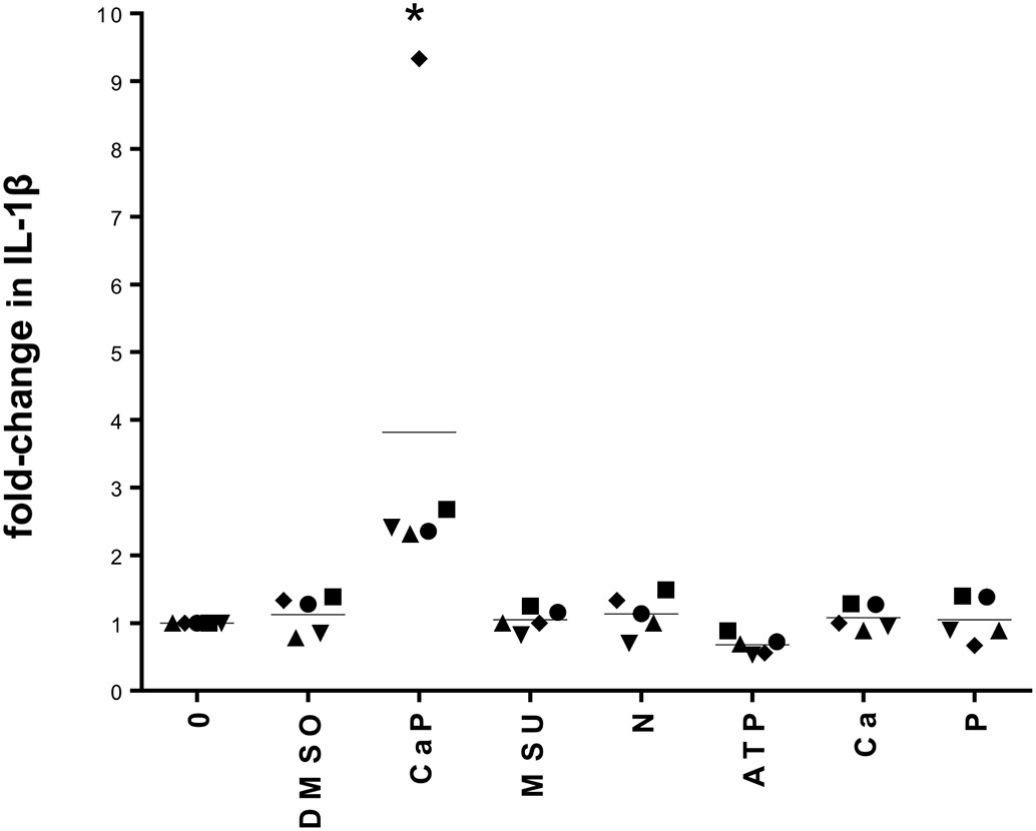
Inflammasome activators MSU, nigericin (N) and ATP do not induce IL-1β release from human VSMCs. ELISA measurement of IL-1β release from 5 different VSMCs isolates (indicated by 5 different symbols), displayed as fold-change related to ‘0’ control values for each cell isolate. Cells were treated with either CaP particles (12.5 μg/mL), MSU crystals (12.5 μg/mL), nigericin (25 μM), ATP (10 mM), CaCl_2_ (5.4 mM, ‘Ca’), Na_2_HPO_4_ (2 mM, ‘P’), vehicle control (DMSO) or no additions (0) for 16 h. Mean levels for each treatment are indicated. Raw data are displayed in Supplement Fig. S4. A one-way ANOVA on log-transformed raw data followed by a Holm-Sidak's multiple comparisons test determined that CaP treatment significantly differed from each of the other treatments (*P < 0.05).

To assess the effects of CaP particles compared with other potential inflammasome activators, VSMCs were incubated with monosodium urate (MSU) crystals, nigericin or ATP. However, none of these stimulated IL-1β release from VSMCs (Fig. 3 and Supplement Fig. S4). Furthermore, exposing cells to high levels of extracellular Ca^2+^ or PO_4_^3−^ did not stimulate IL-1β release (Fig. 3 and Supplement Fig. S4).

### 3.2 CaP particles induce IL-1β release via caspase-1

**Fig. 4:**
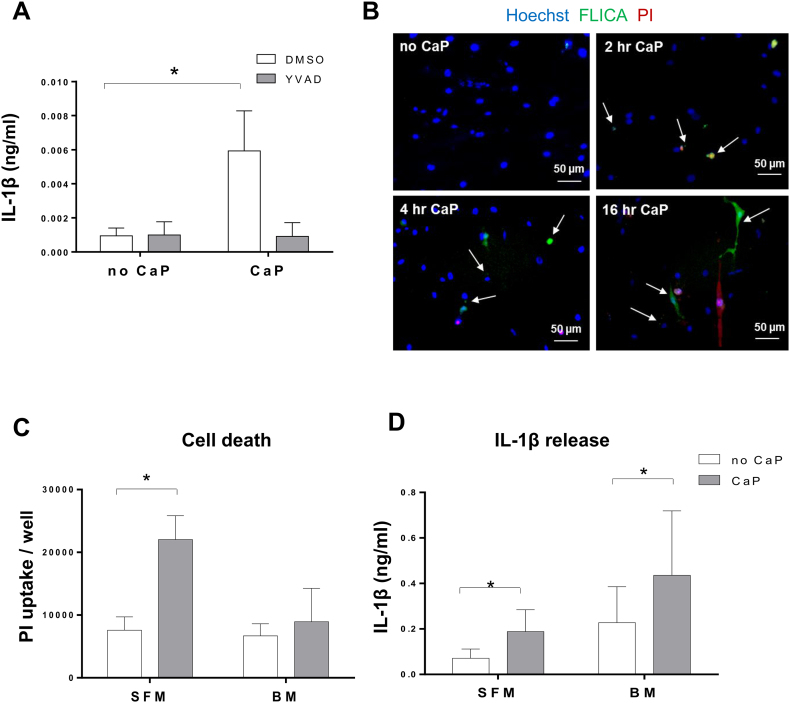
CaP particles stimulate caspase-1 activity. A. ELISA measurement of IL-1β release from 3 different VSMC isolates over 16 h with or without CaP particles (12.5 μg/mL) and with or without YVAD (20 μM). A two-way ANOVA followed by Holm-Sidak's multiple comparisons test was used to determine differences between means. Results are presented as mean ± SEM, *P < 0.05. B. VSMCs were treated with or without CaP particles at indicated times then labelled with FAM-YVAD-FLICA (active caspase-1 reagent), PI and Hoechst and imaged live. Arrows indicate foci/cells containing active caspase-1. Images are representative of 5 experiments in different cell isolates, quantitation of active caspase-1 and PI in live cells is displayed in Fig. 6D and Supplement Figs. S8 and S9. C and D. PI uptake (C) or IL-1β release (D) from 3 different VSMCs isolates in serum-free conditions (SFM) or in basal medium containing 5% FBS (BM) with or without CaP particles (12.5 μg/mL) over 16 h. Mean values ± SEM are displayed. In C, a two-way ANOVA followed by Holm-Sidak's multiple comparisons test was performed and significant differences between corresponding no CaP and CaP-treated samples are indicated by * (P < 0.05). In D, a two-way ANOVA was performed on log-transformed data followed by Holm-Sidak's multiple comparison's test and significant differences between corresponding no CaP and CaP-treated samples are indicated by * (P < 0.05).

Stimulation of IL-1β release from VSMCs by CaP particles was inhibited by treatment with the caspase-1 inhibitor YVAD (Fig. 4A). Live cell imaging of active caspase-1 using a fluorogenic caspase-1 probe (FAM-YVAD-FLICA) revealed active caspase-1 in a low percentage of VSMCs after exposure to CaP particles (Fig. 4B, and for quantitative analysis see Fig. 6D and Supplement Figs. S8 and S9). Active caspase-1 was detected either as a single complex, similar to observations of single inflammasome foci seen in macrophages [39], or in a diffuse pattern. Thus, CaP particles stimulated activation of caspase-1 in a small proportion of VSMCs.

A minority of VSMCs contained PI after CaP particle exposure, indicating that some cells underwent necrosis (Fig. 4B and for quantitative analysis Supplement Figs. S8 and S9). We therefore tested if conditions that enhanced necrosis affected CaP particle-induced IL-1β release. In the absence of serum, CaP particles induced higher levels of necrosis than when serum was present (Fig. 4C), consistent with our previous studies [7]. However, serum-free conditions did not enhance the ability of CaP particles to stimulate IL-1β release, despite higher levels of necrosis (Fig. 4D). These results suggest that necrosis is not the main mechanism involved in CaP particle-induced IL-1β release and that serum factors enhance IL-1β release.

We investigated the potential roles of ROS and lysosomal damage in CaP particle-induced IL-1β release, but found that the ROS scavenger Trolox and the pan-cathepsin inhibitor e64 had no effect on IL-1β release (Supplement Fig. S5 and S6).

**Fig. 5:**
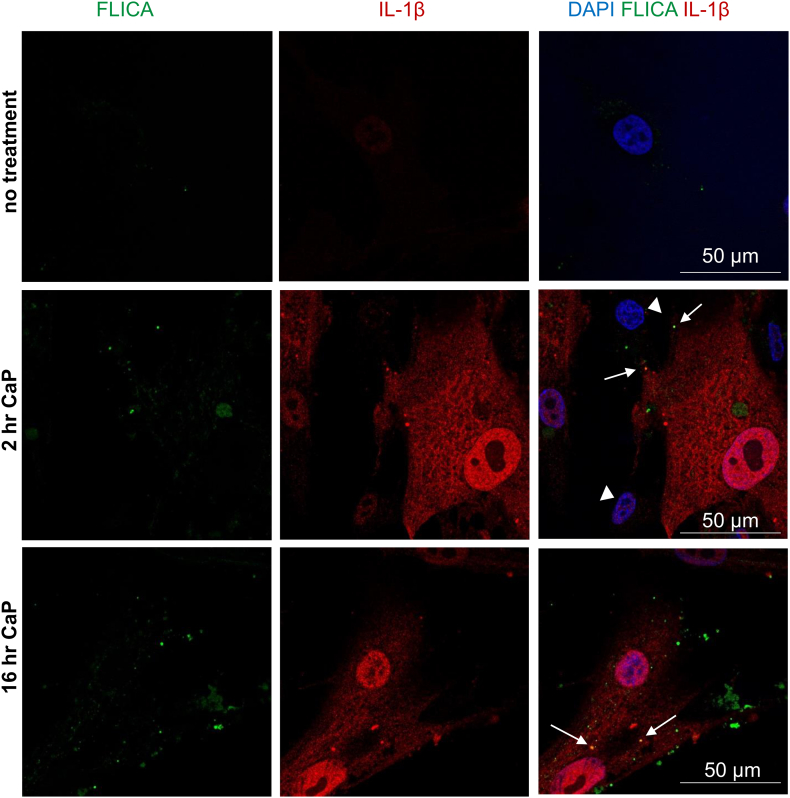
Caspase-1 activity colocalises with IL-1β. VSMCs were treated with or without CaP particles (12.5 μg/mL) for indicated times, labelled with FAM-YVAD-FLICA (active caspase-1 reagent), then fixed and incubated with antibodies recognising IL-1β. Arrows indicate areas in merged images where active caspase-1 and IL-1β overlap. Arrowheads indicate some cells treated with CaP particles that did not contain IL-1β. Confocal images are representative of experiments in 3 different cell isolates.

Using confocal analysis to image active caspase-1 and IL-1β, some areas of colocalisation were observed within VSMCs (Fig. 5), suggesting that active caspase-1 and IL-1β interact after CaP particle stimulation. The colocalisation foci resembled endosomes, both in the perinuclear area and cell periphery.

### 3.3 SYK phosphorylation is required for caspase-1 activation and IL-1β release

**Fig. 6:**
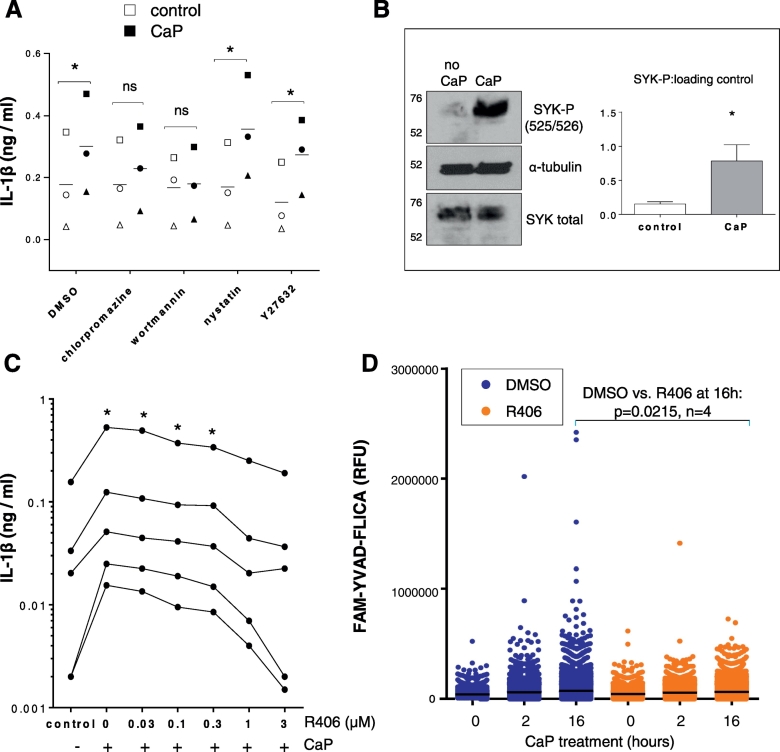
CaP uptake and SYK phosphorylation. A. ELISA measurement of IL-1β release from VSMCs that were exposed to chlorpromazine (1 μg/mL), wortmannin (100 ng/mL), nystatin (10 μg/mL), Y27632 (10 μM) or vehicle control (DMSO) for 1 h, then exposed to CaP particles (12.5 μg/mL) for 16 h. A two-way ANOVA followed by Sidak's multiple comparisons test was used to compare groups. Significant differences are indicated by * (P < 0.01), n = 3, i.e. VSMC isolates from 3 different individuals (indicated by 3 different symbols). B. VSMCs were treated with or without CaP particles (12.5 μg/mL) for 10 min and analysed for SYK or phosphorylated SYK (525/526) by Western analysis. Western blots shown are representative of experiments from 3 different cell isolates (see Supplement Fig. S7) Ratios of SYK-P to loading control were higher in CaP-treated cells, compared with non-treated controls (paired *t*-test of log-transformed data, P < 0.01, n = 3 different isolates). C. ELISA measurement of IL-1β release from 5 different VSMC isolates exposed to CaP particles (12.5 μg/mL) and R406 at concentrations indicated or vehicle control (DMSO). One-way ANOVA of log-transformed data was performed followed by Holm-Sidak's multiple comparisons tests; indicated treatments differed significantly from control values, *P < 0.05. D. Quantitation of active caspase-1 in live VSMCs after treatment with CaP particles (12.5 μg/mL) for 0, 2 or 16 h with either a 1 h pre-treatment with R406 (1 μM) or equivalent DMSO. Results shown are representative of 4 independent experiments in different cell isolates. Scatter plots display fluorescence levels of the caspase-1 substrate (FAM-YVAD-FLICA) for approximately 10,000 live VSMCs for each treatment group. Each dot represents the active caspase-1 level for 1 cell. As indicated, when comparing VSMCs from 4 different individuals, CaP particles increased active caspase-1 activity and this effect was reduced in the presence of R406, when comparing the top 5th percentile of VSMCs at the 16 h time point (P = 0.0215).

Next, we investigated potential signalling mechanisms activated by CaP particles upstream of caspase-1. We have previously shown by electron microscopy that CaP particles are rapidly endocytosed by VSMCs [8]. Inhibitors of endocytosis, wortmannin and chlorpromazine, reduced IL-1β release, while nystatin and Y27632 did not reduce IL-1β levels (Fig. 6A). These results suggest that clathrin-mediated endocytosis was involved in particle uptake and IL-1β release.

As SYK activation has been linked with crystal-membrane interactions, endocytosis and inflammasome activation, we investigated SYK activation after CaP particle exposure. CaP particle treatment did not change SYK protein levels but increased phosphorylation at Y525/Y526, in the SYK activation loop (Fig. 6B and Supplement Fig. S7A and B). In addition, the SYK inhibitor R406 reduced CaP particle-induced IL-1β release in a concentration-dependent manner (Fig. 6C). Full inhibition of CaP-induced IL-1β release was observed with R406 (1 or 3 μM), and this was the case for each VSMC isolate tested, regardless of whether cells had low or high levels of basal IL-1β release. Furthermore, R406 inhibited caspase-1 activity after CaP particle exposure, as assessed by quantitative live cell imaging (Fig. 6D), with no effect on VSMC death (Supplement Fig. S8 and S9). This suggests that SYK activation occurs prior to and is required for caspase-1 activation and thence IL-1β in response to CaP particles.

### 3.4 Exosomes as a route for IL-1β release

**Fig. 7:**
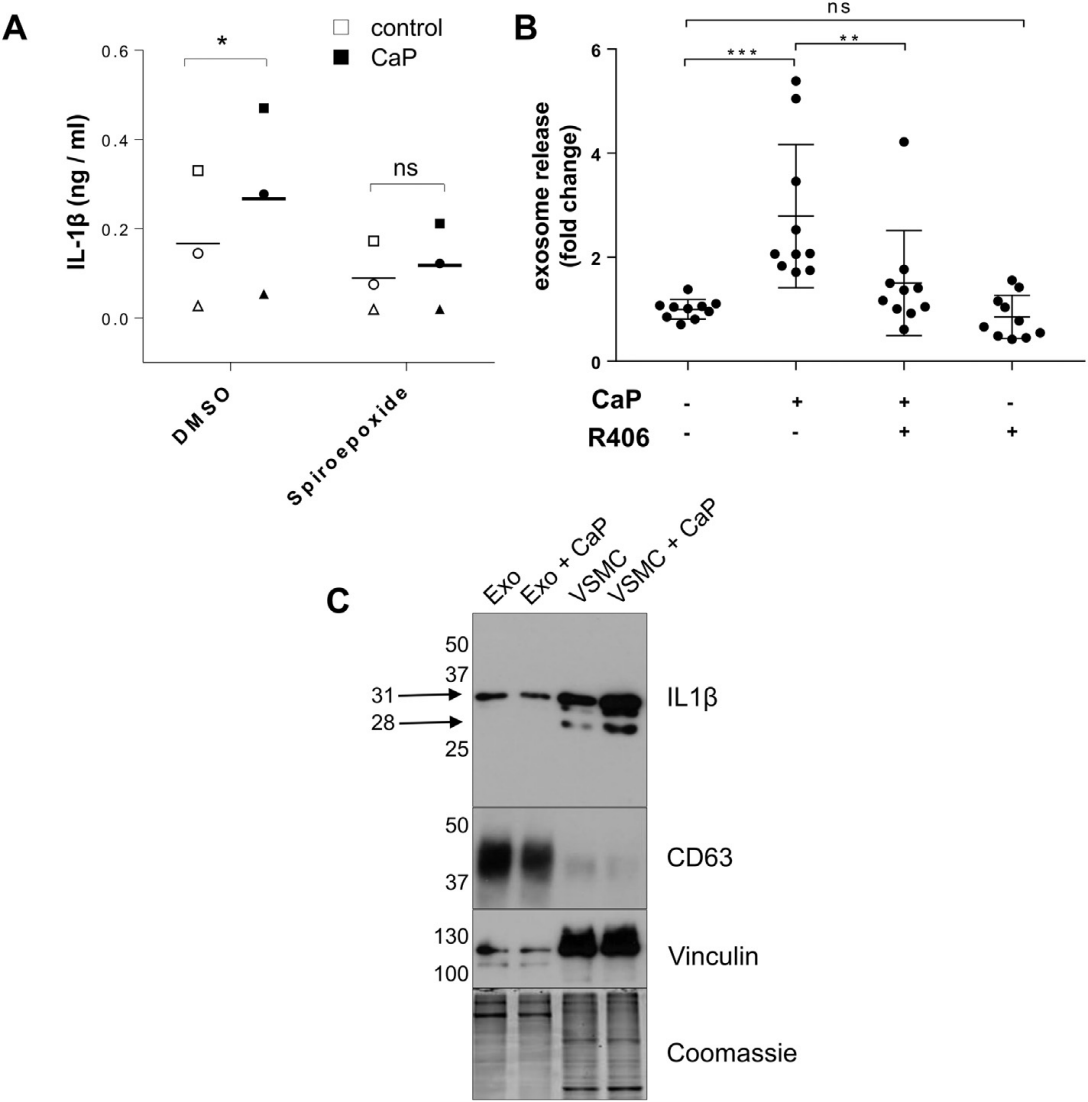
Release of IL-1β via exosomes. A. VSMCs were treated with or without CaP particles (12.5 μg/mL) for 16 h with or without a pre-treatment with spiroepoxide (10 μM). A two-way ANOVA followed by Sidak's multiple comparisons test was used to compare groups. Significant differences are indicated by * (P < 0.05), n = 3, i.e. VSMC isolates from 3 different individuals (indicated by 3 different symbols). B. Exosome secretion by VSMCs. VSMCs were incubated in 2.5%FBS/M199 media with or without CaP particles (12.5 μg/mL) and with or without R406 (1 μM) for 16 h. Conditioned media were harvested and exosomes were quantified using an anti-CD63 bead-capturing assay which detects CD63/CD81 exosomes. Statistical significance was tested by one-way ANOVA followed by Sidak's multiple comparisons test. Significant differences are indicated by **(P < 0.01), ***(P < 0.001), n = 10. C. Western analysis of exosomes isolated by differential ultracentrifugation from conditioned media of VSMCs treated with or without CaP particles for 16 h. Exo, exosomes; VSMC, whole cell lysates. The membrane was probed with IL-1β antibodies (R and D), CD63 (BD Pharminigen), with vinculin [38] and Coomassie brilliant blue staining to demonstrate protein loading.

To assess whether vesicle release may be a mechanism for IL-1β release from VSMCs, cells were incubated with an exosome release inhibitor, spiroepoxide [38]. Spiroepoxide reduced CaP particle-induced IL-1β release, suggesting that exosomes are involved in the release of IL-1β (Fig. 7A). We next measured exosome release from VSMCs in response to CaP particles and found that CaP particles stimulated exosome release (Fig. 7B). Additionally, the SYK inhibitor R406 inhibited CaP particle-induced exosome release, suggesting that SYK phosphorylation is involved in CaP particle-stimulated exosome release (Fig. 7B).

To confirm if IL-1β is secreted in exosomes, we isolated exosomes from conditioned media of VSMCs treated with or without CaP particles. We previously showed that VSMC exosomes are enriched with tetraspandin CD63 [38] and correspondingly, Western blot analysis revealed that exosomes enriched with CD63 contained the pro-form of IL-1β (31 kDa) (Fig. 7C). Notably, CaP particle-treated and control cells released exosomes with similar levels of IL-1β, which suggests that the increase in released IL-1β detected by ELISA after CaP particle treatment may be due to increased numbers of released vesicles, rather than selective loading of released exosomes with IL-1β.

The mature, active, 17 kDa form of IL-1β was not detected in cells or exosomes but a 28 kDa band was observed in cells in addition to the 31 kDa pro-IL-1β (Fig. 7C, Supplement Fig. S2B). The 28 kDa form of IL-1β has been reported in other studies as an intermediate form, which is generated upon the first step in caspase-1-mediated cleavage of pro-IL-1β [40]. Although we found active caspase-1 at foci within cells, indicating intracellular caspase-1 activity, active caspase-1 was also detected in cell supernatants by Western Blotting (Supplement Fig. S10), suggesting that some IL-1β processing may also occur extracellularly.

## 4 Discussion

Here we report that CaP particles induce the release of IL-1β from human VSMCs via activation of SYK and caspase-1. The levels of basal and CaP particle-stimulated IL-1β release varied between VSMCs isolated from different donors and also with stage of cell culture; from early passage cells, where IL-1β levels were lowest, to cells that had undergone replicative senescence, where IL-1β levels were highest. This suggests that senescent VSMCs exposed to CaP particles have the greatest pro-inflammatory potential, compared with proliferating cells, which is particularly relevant to atherosclerotic plaque caps where VSMCs that have undergone senescence are thought to weaken plaques and encourage plaque instability, in aged populations in particular [41], [42]. Why VSMCs are already ‘primed’ to express pro-IL-1β is not known but may be related to their de-differentiated, non-contractile phenotype in culture that is commonly observed in diseased blood vessels in vivo. Various mechanisms have been proposed for the basal, unstimulated cytokine release by senescent VSMCs, including altered signalling pathways and genomic instability [28], [29], [43], [44].

Our studies revealed that human VSMCs do not show enhanced release of IL-1β in response to several of the common inflammasome agonists reported to activate caspase-1 in macrophages, including ATP, nigericin and MSU crystals. This suggests that either: (1) human VSMCs do not share the same upstream inflammasome-activating mechanisms as other cell types (e.g. VSMCs lose purinergic receptor expression in culture [45]), and that CaP particle composition or physical parameters such as shape, size and charge may be important in inflammasome activation in VSMCs; or (2) that CaP particles may have additional stimulatory effects on release mechanisms for IL-1β. Potential dissolution of the particles being responsible for the effects on IL-1β release was unlikely, since elevated extracellular levels of either Ca^2+^ or PO_4_^3−^ did not mimic the effects of CaP particles. Thus, VSMCs appeared to be particularly sensitive to CaP particles in terms of stimulating IL-1β release.

Using live cell imaging, active caspase-1 was detected in a minority of VSMCs exposed to CaP particles, perhaps reflecting transient activity of caspase-1. Alternatively, since VSMCs are well known to be heterogeneous, certain phenotypes within the population of cells may be intrinsically primed to activate caspase-1 in response to CaP particles. Further investigation including phenotyping of cells would help to elucidate why some VSMCs have higher potential for activating caspase-1. The finding of high levels of active caspase-1 in subsets of cells and the assumption that IL-1β is activated within these cells may explain why we observed relatively low levels of IL-1β release from VSMCs (mostly in the picomolar range), compared with other cell types such as macrophages, where agonists activate caspase-1 in almost the whole cell population, depending on the potency of the stimulus [46]. The different primary cell isolates used in the current study were from 8 healthy donors (4 males and 4 females) and in general, we found that responses to CaP particles were similar between males and females. However, as we used a relatively small number of donors, this is a limitation of the study. It would be interesting to investigate if CaP particles stimulated caspase-1 activation and IL-1β release at higher levels or in the majority of VSMCs derived from a larger cohort of aged or diseased blood vessels. A recent study of over 100 individuals identified a sub-group of older people with hypertension that had elevated inflammasome gene expression profiles and constitutive IL-1β expression [47]. Since calcification is closely linked with hypertension, aging and cardiovascular mortality [48], it would be particularly interesting to investigate caspase-1 activation and IL-1β release from VSMCs derived from hypertensive patients.

Having established that IL-1β was released by VSMCs in response to CaP particles, we sought to establish which mechanisms control its release. IL-1β is a leaderless protein and is not secreted by the classical secretory pathway. Several mechanisms for its release have been proposed including microvesicle shedding from the plasma membrane, secretion via exosomes, exocytosis of secretory lysosomes, secretion across the plasma membrane during pyroptotic death, secretory autophagy and passive loss during necrosis [49], [50], [51]. Confocal images of VSMCs suggest IL-1β may be released via vesicle-like structures, most likely late endosomes/multivesicular bodies, and this concept was supported by the finding that inhibition of exosome secretion reduced CaP particle-induced IL-1β release. It is also possible that necrosis contributed to the release of IL-1β, since PI positive cells were detected after 2 h of treatment with CaP particles. However, in serum-free conditions where levels of necrosis were higher than when serum was present, CaP particle-induced IL-1β levels were found to be lower, arguing against a necrosis-dependent mechanism causing IL-1β loss. The reason for lower IL-1β release in serum-free conditions could be due to several mechanisms such as: [1] serum being required for efficient IL-1β release; [2] because of cell death, fewer cells are available to respond to CaP particles and activate signalling leading to IL-1β release; or [3] that cells entering quiescence release less IL-1β. However, the latter possibility is less likely as we observed that highly proliferative cells from very early passages released the lowest levels of IL-1β. Additionally, we found that SYK inhibition reduced caspase-1 activation but did not reduce numbers of PI-positive cells, suggesting different mechanisms for CaP particle-induced activation of SYK and CaP particle-induced necrosis. Furthermore, quantitative analysis of PI levels and caspase-1 levels in VSMCs revealed that cells with high PI (dead cells) had relatively low levels of active caspase-1, suggesting that pyroptosis is not involved. However, the kinetics and extent of CaP particle damage to VSMCs and its relation to SYK activation require further investigation, especially in regard to studies reporting that slow-onset necrosis stimulates the NLRP3 inflammasome, while rapid necrosis does not [52]. Our results suggest that in an environment with low serum, CaP particles will induce substantial VSMC death and IL-1β release, while in a serum-containing environment VSMC death is negligible, but signalling leading to IL-1β release is greater. In either scenario, inflammation is the expected result.

SYK activation is known to be linked with crystal-plasma membrane interactions and endocytosis, and may have a large range of effects on downstream signalling such as PI3K and Akt activation [53], [54], [55], [56]. We previously found that CaP particles are taken up by VSMCs via endocytosis or macropinocytosis within 5–10 min of exposure to CaP particles, as demonstrated by electron microscopy [8] and here we found that inhibition of clathrin-mediated endocytosis reduced the effects of CaP particles on IL-1β release. This suggested that particle uptake, rather than membrane surface activation, is important in initiating signalling leading to IL-1β release. However, since endocytosis inhibitors such as wortmannin can have off-target effects, further studies are required to investigate the mechanisms involved in the endocytosis of CaP particles and how this might lead to SYK activation. SYK was activated rapidly in VSMCs, consistent with kinetics of activation reported in other cell types [57] and the highly selective SYK inhibitor, R406, inhibited CaP-induced IL-1β release. SYK is involved in various biological processes including focal plasma membrane damage-induced necroptosis in red blood cells [58], while overexpression of SYK in tumor cells leads to senescence [59]. However, it appeared that in VSMCs, SYK activation, rather than raised SYK protein levels, was important in IL-1β release. Inhibition of SYK phosphorylation with R406 also inhibited caspase-1 activation, indicating that SYK phosphorylation occurs upstream of caspase-1 and is required for caspase-1 activation. It will be interesting in future studies to investigate the intermediate steps that link SYK phosphorylation to caspase-1 activation, such as other kinases and which inflammasome is involved.

We found that the highly selective inhibitor of neutral sphingomyelinase, spiroepoxide, which inhibits exosome release, inhibited CaP particle-induced IL-1β release. Further investigation found that CaP particles stimulated exosome release, suggested that IL-1β may be exported from VSMCs via exosomes. Supporting this hypothesis, we found that R406 inhibited CaP particle-induced exosome release and that exosomes contained IL-1β. SYK involvement in exosome release has been suggested by others [60] and raises the possibility that R406 inhibits IL-1β release by more than one mechanism, i.e. inhibition of caspase-1 activation and inhibition of exosome release. We demonstrated a role for exosomes in IL-1β release but secretion via other extracellular bodies and/or microvesicles cannot be excluded and require further investigation. Exosome release is known to be increased in senescent cells, which may help to explain why higher basal release of IL-1β was observed in senescent VSMCs. In conditions such as those found in the environment of the atherosclerotic plaque, exosomes released from VSMCs have been shown to initiate and propagate mineralisation [38]. Thus, a reduction in exosome release by R406 would also be expected to reduce calcification, although this remains to be tested experimentally.

Intriguingly, the mature, active, 17 kDa form of IL-1β was detected in VSMC supernatants but not in cells or exosomes. The pro-form of IL-1β (31 kDa) and an additional band at 28 kDa was observed. A lack of detection of mature IL-1β may have been due to low levels in cells or exosomes, or perhaps due to rapid release of active IL-1β. The presence of a 28 kDa form of IL-1β has been described as an intermediate, first step in caspase-1 cleavage [40], [61]. Thus, IL-1β was detected intracellularly in VSMCs in a pro- and intermediate cleaved form, in exosomes in a pro-form, while IL-1β in its pro-, intermediate cleaved and mature form was detected in cell supernatants. It should be noted that levels of cleaved forms of IL-1β were low, relative to pro-IL-1β levels. This observation raises the question of how and where IL-1β is cleaved to generate the mature form. Clearly, the initial step in pro-IL-1β cleavage was detected within cells and the presence of active caspase-1 within foci in VSMCs suggests intracellular caspase-1 activity. However, active caspase-1 was also found in cell supernatants, raising the possibility that some IL-1β processing may occur extracellularly in VSMC cultures. Although most studies in macrophages describe IL-1β cleavage by caspase-1 within cells before IL-1β is released, the cleaved form of caspase-1 is often detected in cell supernatants [56], [62] and a recent study highlighted the detection of extracellular active caspase-1 released from human monocyte/macrophages stimulated with LPS and ATP [63]. The idea of extracellular processing of pro-IL-1β is not new and other proteases, such as neutrophil and mast cell-derived proteases have been implicated in pro-inflammatory environments (reviewed in [61]). This highlights that even if inactive forms of IL-1β are released by cells, they may be important in generating active IL-1β in vivo. Additionally, several studies have reported that exosomes were implicated in the transfer of fully functional cytokines and growth factors [64], [65], [66]. Further studies are required to determine how IL-1β is packaged and released from VSMCs, and to what extent it is functional.

Mature IL-1β is a potent cytokine known to stimulate neutrophil migration, cause vascular permeability and induce further calcification, all important disease-amplifying mechanisms. Blockade of IL-1β activation in atherosclerotic plaques is desirable, as reported in a recent clinical trial that successfully targeted IL-1β and reduced cardiovascular event rates [67]. The SYK inhibitor used in this study, R406 is the orally active metabolite of fostamatinib, which is currently in clinical trials for autoimmune thrombocytopenia, haemolytic anaemia and IgA nephropathy. In previous clinical trials fostamatinib was found to be ineffective in treating rheumatoid arthritis but it was well tolerated. In a mouse model of atherosclerosis, fostamatinib was effective in reducing atherosclerotic lesion size and macrophage infiltration [68]. Thus, SYK inhibition may have therapeutic potential in the vasculature and perhaps also at other locations where CaP particles appear to be damaging, such as in osteoarthritis.

In conclusion, our studies in primary human VSMCs suggest that CaP particles activate SYK and caspase-1 leading to IL-1β release via exosomes. If similar mechanisms occur in vivo, inhibition of SYK could potentially interfere with the damaging effects of microcalcification in VSMC-rich fibrous caps in addition to other proinflammatory targets in the diseased blood vessel wall. Further work is now required to decipher: how CaP particles activate SYK in VSMCs; exactly how this leads to caspase-1 activation, exosome and IL-1β release; and to explore the importance of CaP particle-induced cytokine release in vivo.

## Funding

This work was funded by the British Heart Foundation (Career Re-entry Fellowship to D.P. FS/11/21/28691 and a project grant to AK PG/17/37/33023) and supported by an Institute Strategic Programme Grant from the Biotechnology and Biological Sciences Research Council (BB/PØ13384/1).

## Appendix A Supplementary data

Supplementary material Image 1
