# Supplementary material for: Calcium phosphate particles stimulate interleukin-1β release from human vascular smooth muscle cells: A role for spleen tyrosine kinase and exosome release
Source: J Mol Cell Cardiol. 2018 Feb;115:82–93. doi: 10.1016/j.yjmcc.2017.12.007 (PMC5823844; doi:10.1016/j.yjmcc.2017.12.007)
Supplement: Supplementary file 1 — Supplementary material [file mmc1.pdf]

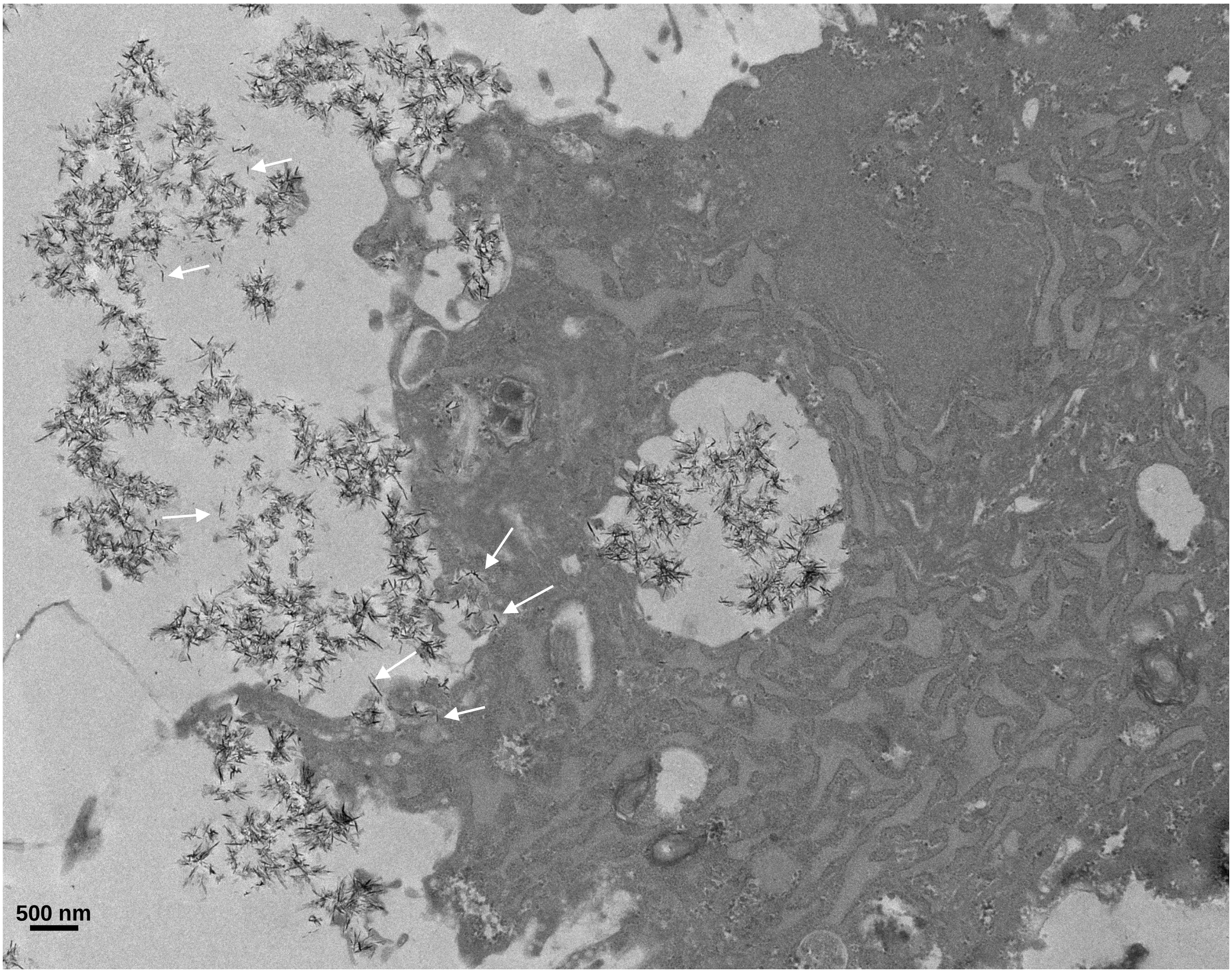

Figure S1. Ultrastructural analysis of CaP particle exposure to human VSMCs
CaP particles (12.5 µg/ml) were incubated with human VSMCs for 10 minutes and subsequently fixed and processed for transmission electron microscopy analysis, as described previously [8]. Note that particles of approximately 200nm agglomerate and some individual particles are observed (indicated by arrows).

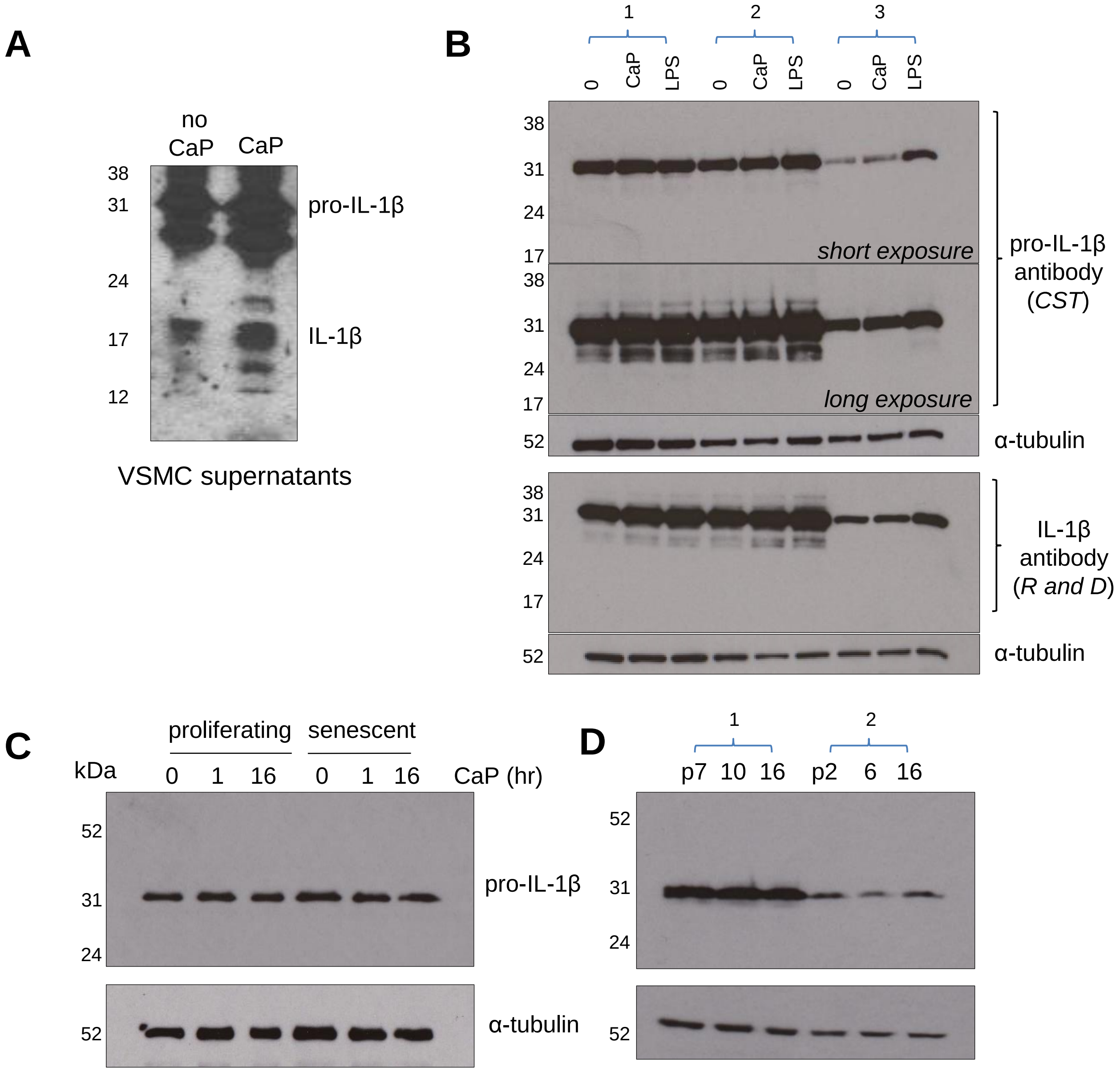

Figure S2. CaP particles do not stimulate pro-IL-1β

A. Western blot of supernatants from VSMCs treated with or without CaP particles (12.5 µg/ml). Supernatants were concentrated by a factor of 10 on a 10 kDa spin column (Amicon). Using an antibody that recognises the mature, 17 kDa form of IL-1β (R and D), a band at 17 kDa was observed in the supernatants, with higher levels in CaP-treated samples. Bands at 31 kDa (pro-IL-1β) and 28 kDa were also observed. **B**. Western analysis of IL-1β levels in VSMC lysates from 3 different individuals treated with or without CaP particles (12.5 µg/ml) or LPS (0.1 µg/ml) for 24 hours. Pro-IL-1β levels were unchanged by CaP particle treatment in each VSMC isolate. With LPS treatment, pro-IL-1β levels were not altered in VSMC isolates #1 and 2, whereas VSMC isolate #3 displayed increased pro-IL-1β in response to LPS. Using either an antibody recognising pro-IL-1β (CST, top 2 blots) or to mature IL-1β (R and D, lower blot), a 17 kDa band was not detected in VSMCs lysates but a 28 kDa band was seen, particularly in CaP and LPS-treated cells. **C**. Pro-IL-1β levels in VSMCs from a different cell isolate to that in Fig 2C with proliferating (mid-passage, p7) or senescent cells (p18) treated without (0) or with CaP particles (12.5 µg/ml) for 1 or 16 hours. **D.** Pro-IL-1β levels in VSMCs from different cell isolates to that in Fig 2C and Supplement 2C at different stages of cell culture: at passage 7, 10 (mid-passage) and 16 (senescent) or 2 (early passage), 6 (mid-passage) and 16 (senescent), indicating no apparent changes in pro-IL-1β levels between early, mid-passage and senescent cultures. C and D were probed with antibodies recognising pro-IL-1β (CST). Blots in B, C and D were re-probed with α-tubulin as a loading control.

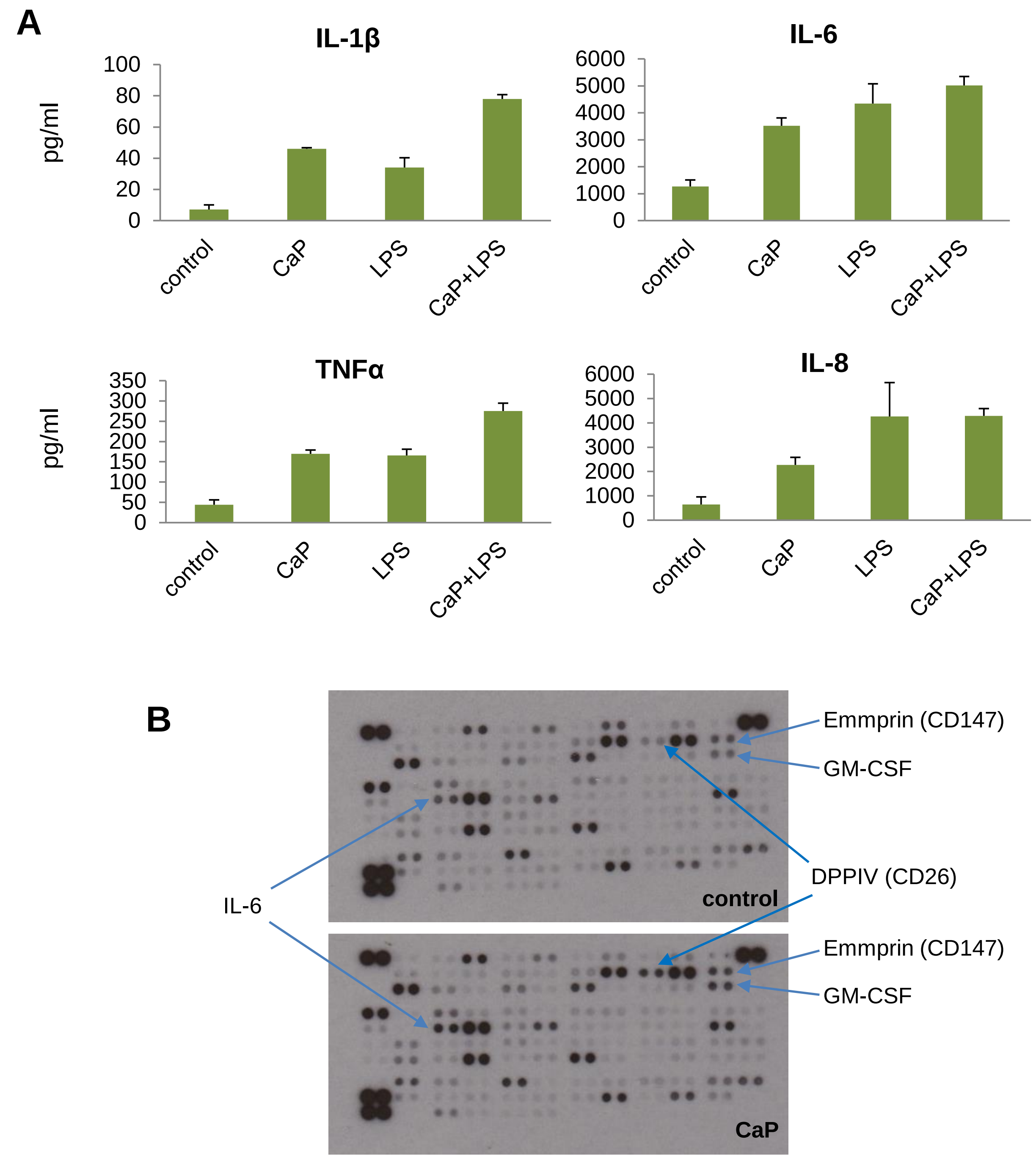

Figure S3. CaP particles stimulate cytokine release from VSMCs.
A. Simultaneous measurement (multiplex ELISA) of IL-1β, TNFα, IL-6 and IL-8 release from human VSMCs over 16 hours with or without CaP particles (12.5 µg/ml) and with or without a 24-hour pre-treatment with LPS (0.1 µg/ml). The control contained equivalent PBS. Results shown are means and s.d. from 3 replicates. These results suggest that CaP particles alone stimulate IL-1β, TNFα, IL-6 and IL-8 from VSMCs.
B. Conditioned medium (supernatants) from VSMCs treated with or without CaP particles (12.5 µg/ml) were exposed to a Human XL Cytokine array containing 105 analytes (R and D, ARY022B), following the manufacturers protocol. Arrows indicate duplicate spots where increased levels of specific cytokines were detected after CaP particle treatment. i.e., DPPIV (CD26), IL-6, GM-CSF and emmprin (CD147). Thrombospondin-1 and BDNF were released at lower levels after CaP particle treatment. Results shown are representative of 2 separate experiments.

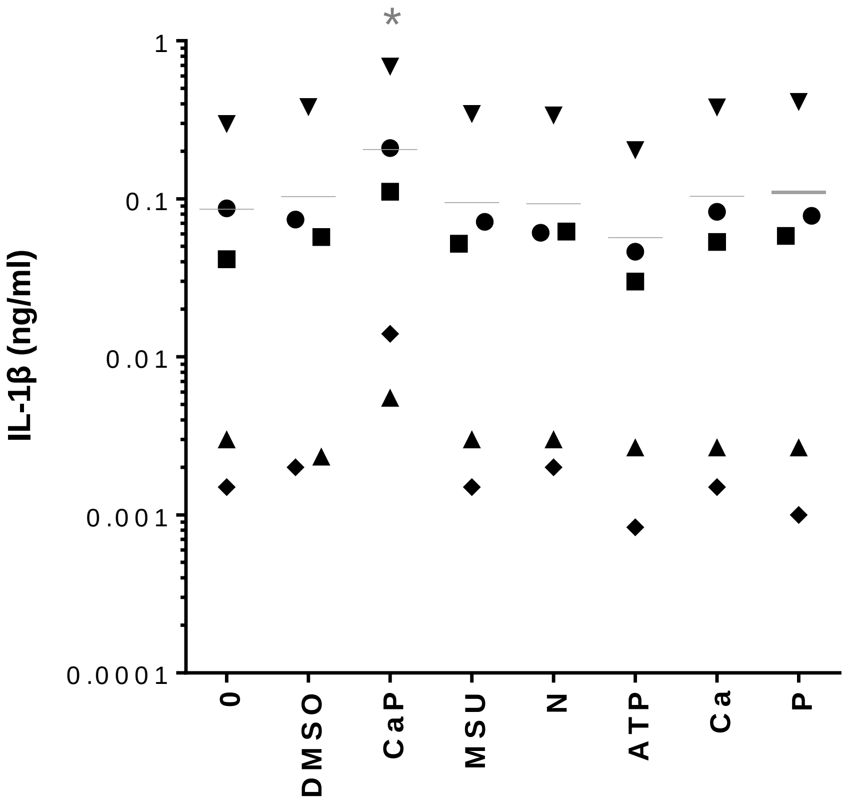

Figure S4. MSU, nigericin (N) and ATP do not induce IL-1β release from human VSMCs.
As for Figure 3 in the main text**,** but raw data are displayed here on a log-scale. ELISA measurement of IL-1β release from 5 different human VSMCs isolates (each isolate indicated by a different symbol). Cells were treated with either CaP particles (12.5 µg/ml), MSU crystals (12.5 µg/ml), nigericin (25 µM), ATP (10 mM), $CaCl_2$ (5.4 mM, 'Ca'), Na_2HPO_4 (2mM, 'P'), vehicle control (DMSO) or no additions (0) for 16 hours. Mean levels for each treatment are indicated. A one-way ANOVA on log-transformed raw data followed by Holm-Sidak's multiple comparisons test determined that CaP particle treatment significantly differed from each of the other treatments (*P<0.05).

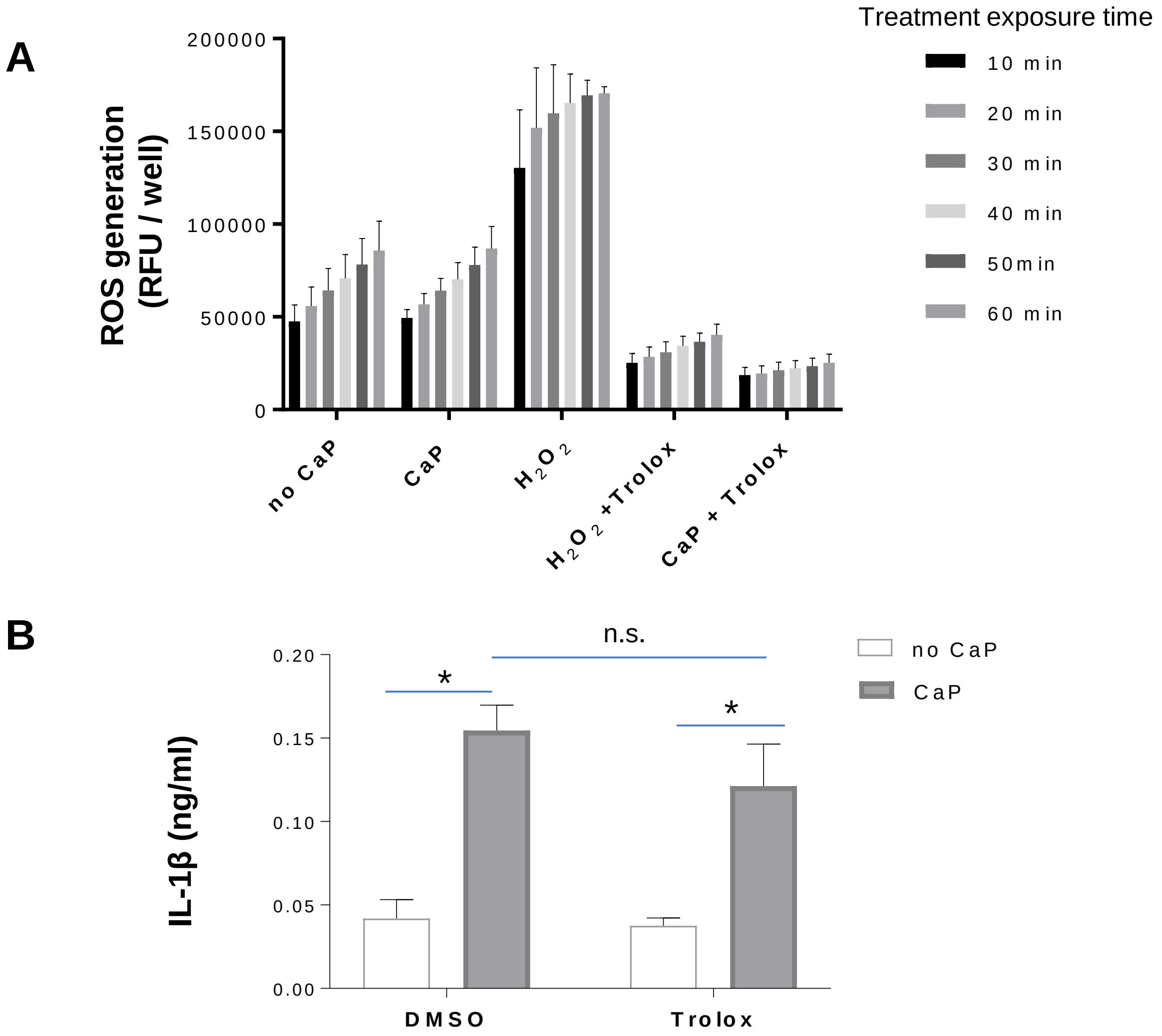

Figure S5.

A. Activation of ROS is not stimulated by CaP particles

ROS measurement after treatment of DCFDA-loaded human VSMCs with CaP particles or H_2O_2. Cells were plated in 96-well plates at 5,000 cells/ well in BM and allowed to attach overnight. Cells were then incubated with 10 µM DCFDA (Sigma) and incubated at 37 ℃ for 30 minutes. Media were then replaced with physiological buffer [7] and treated with CaP particles (12.5 µg/ml), or 1 mM H_2O_2 in the presence or absence of a ROS scavenger Trolox (100 µM) for times indicated. Plates were read on a Pherastar fluorescence plate reader (495 excitation/529 emission, BMG Labtech) and fluorescence units were analysed relative to background counts (cells with no DCFDA). Results are presented as means ± s.d. (n = 4).

B. Activation of ROS does not appear to be involved in CaP-induced IL-1β release

ELISA measurement of IL-1β release from human VSMCs treated with either CaP particles (12.5 µg/ml), vehicle control (DMSO) or Trolox (100 µM) for 16 hours. A two-way ANOVA on log-transformed raw data followed by Holm-Sidak's multiple comparisons test determined that CaP particle treatment significantly differed from no CaP control treatments (*$P<0.05$), but that the reduction in CaP-induced IL-1β release observed in the presence of Trolox was not significant ($P>0.05$, n = 3).

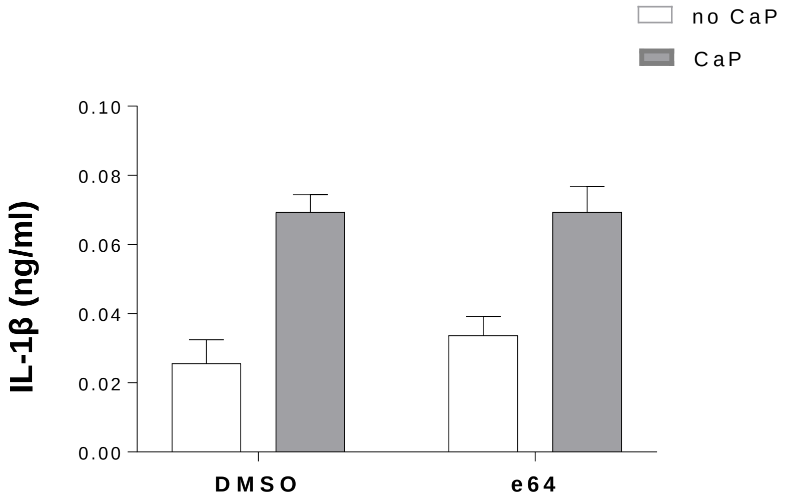

Figure S6. The lysosomal cathepsin inhibitor e64 does not affect CaP-induced IL-1β release
ELISA measurement of IL-1β release from human VSMCs treated with either CaP particles (12.5 µg/ml), vehicle control (DMSO) or e64 (10 µM, Sigma)
for 16 hours. E64 did not affect CaP particle-induced IL-1β release (n = 3).

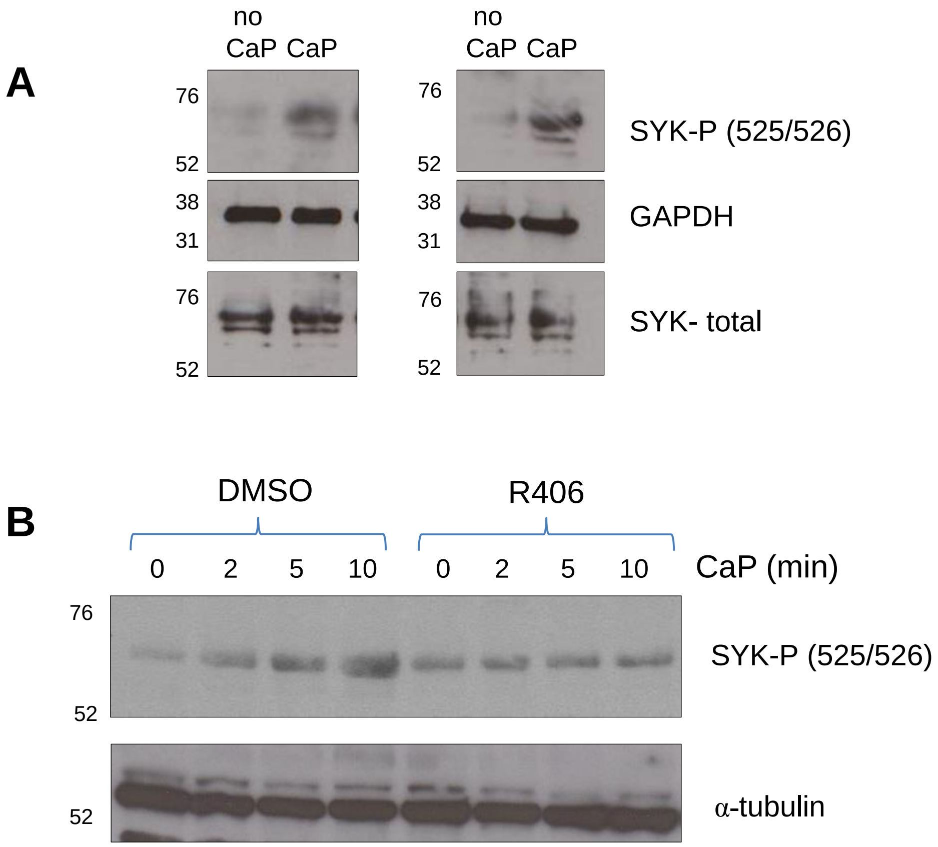

Figure S7.
A. As for Figure 6B in the main text but results from cells derived from 2 different individuals are displayed. Cells were treated with or without CaP particles for 10 minutes and analysed for SYK expression by Western analysis using antibodies raised to SYK or phosphorylated SYK (525/526), with GAPDH as the loading control (directly re-probing the SYK-P blot). Blots show that SYK-P levels are increased in CaP particle-treated cells.
B. Cells were treated with or without CaP particles for indicated durations, with a 1-hour pre-treatment with either vehicle (DMSO) or R406 (1 µM) and analysed for SYK-P expression by Western analysis as above with α-tubulin as the loading control. The blot shows that SYK-P levels were increased in CaP particle-treated cells and this effect was inhibited in the presence of R406, i.e. confirming that R406 inhibits SYK phosphorylation (525/526).

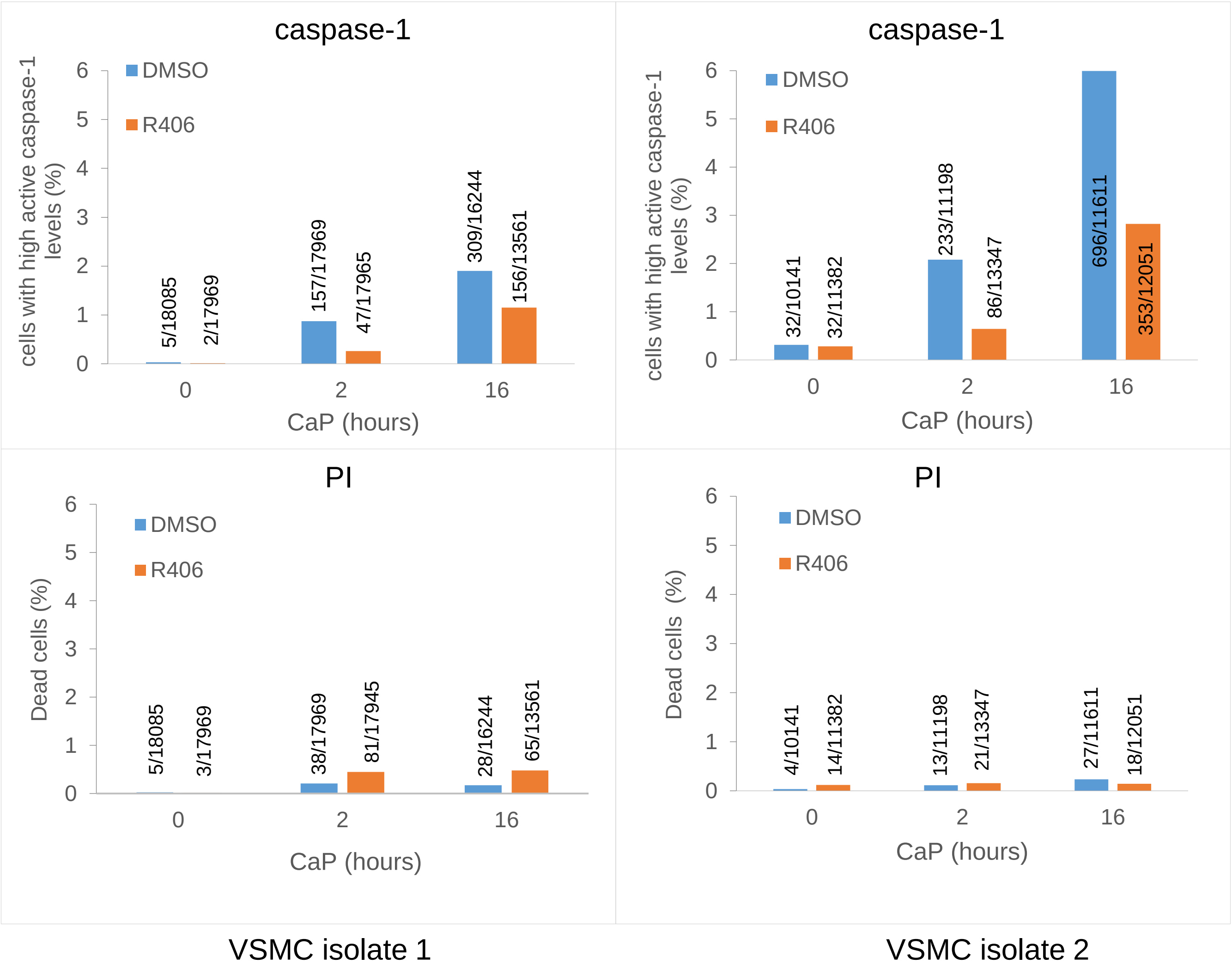

Figure S8. R406 reduces CaP particle-induced caspase-1 activity but not cell death
Quantitation of active caspase-1 in live VSMCs from 2 different individuals after treatment with CaP particles (12.5 µg/ml) for 0, 2 or 16 hours with either a 1 hour pre-treatment with R406 (1 µM) or equivalent DMSO. The percentage of cells expressing high levels of active caspase-1 (top panels) and PI uptake (lower panels) are shown. Thresholds were set using untreated controls (0 CaP) fluorescence values to determine numbers of cells expressing 'high' caspase-1 or PI positive cells. The same threshold values were used for both VSMC isolates. Raw data used to determine percentage values are shown for each treatment. A trend was observed where CaP particles increased the percentage of cells displaying high active caspase-1 activity, an effect that was reduced in the presence of R406. R406 treatment did not show the same trend on PI uptake (cell death) in VSMCs, i.e. R406 reduced active caspase-1 levels in response to CaP particles but did not reduce cell death.

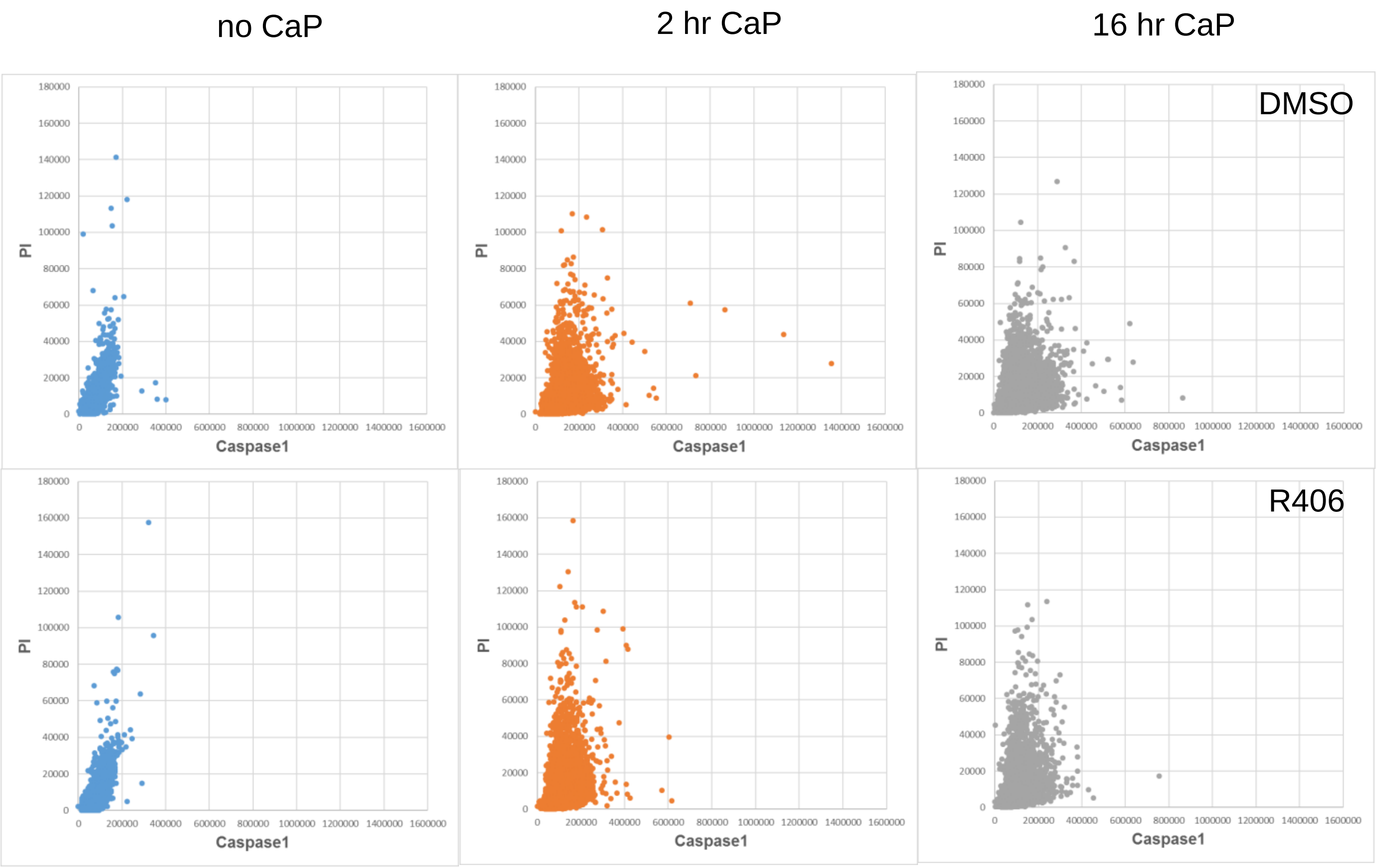

Figure S9. R406 reduces CaP particle-induced caspase-1 activity but not cell death
Quantitation of active caspase-1 in live VSMCs after treatment with CaP particles (12.5 µg/ml) for 0, 2 or 16 hours with either a 1 hour pre-treatment with R406 (1 µM) or equivalent DMSO. CaP particles increased the number of cells displaying high active caspase-1 activity, an effect that was reduced in the presence of R406, seen here as a shift to the left with R406 treatment. R406 treatment did not show the same trend on PI uptake (cell death) in VSMCs, i.e. R406 reduced active caspase-1 levels in response to CaP particles but did not reduce cell death. Note that cells with the highest PI levels (dead cells) had relatively low active caspase-1 levels. Results shown are representative of 2 independent experiments.

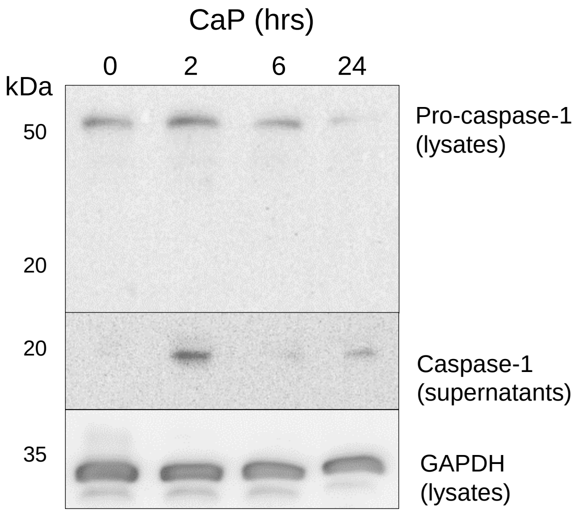

Figure S10. Detection of active caspase-1 in VSMC supernatants
Western analysis of VSMC lysates or supernatants treated with CaP particles (12.5 µg/ml) for indicated times. The caspase-1 antibody used recognises both mature caspase-1 and cleaved caspase-1 (20 kDa, Adipogen). The 20 kDa, active form of caspase-1 was detected only in cell supernatants, possibly due to rapid release from cells and accumulation extracellularly. Cell lysate blots were re-probed with GAPDH as a loading control.
